# Particle Retention Capacity, Efficiency, and Mechanism of Selected Plant Species: Implications for Urban Planting for Improving Urban Air Quality

**DOI:** 10.3390/plants10102109

**Published:** 2021-10-05

**Authors:** Huixia Wang, Hui Shi

**Affiliations:** School of Environmental and Municipal Engineering, Xi’an University of Architecture & Technology, Xi’an 710055, China; dasenlin-80@163.com

**Keywords:** particulate matter, retention efficiency, different scales, urban planting, air quality improvement

## Abstract

Atmospheric particulate matter (PM) has been of concern owing to its negative effects on human health and its role in environmental degradation. For mitigation purposes, it is important to select the most efficient plant species in urban greening. Here, a fast, cost-saving methodology was first added to the conventional method to investigate the size-resolved PM retention capacity and efficiency of twenty plant species. Surface PM (SPM), which can be removed by water and brushing, accounted for 44.9–66.9% of total PM, in which the water-soluble PM (DPM) accounted for 12.9–22.1% of total PM. A large mass proportion of in-wax PM (14.1–31.7%) was also observed. *Platycladus orientalis*, *Eriobotrya japonica*, *Viburnum odoratissimum*, *Magnolia grandiflora* had the highest *AE_leaf_* (retention efficiency on per unit leaf area) to retain SPM within different diameter classes (DPM, PM_0.1–2.5_, PM_2.5–10_, PM_>10_). *AE_plant_* (retention efficiency of individual tree) varied greatly among different plant species, mainly due to the dependence on the total area of a tree. *AE_land_* (retention efficiency on per unit green area) is a suitable index for PM retention ability and efficiency. In general, *P. orientalis*, *V. odoratissimum*, *Pittosporum tobira*, *Photinia serrulate*, *M. grandiflora*, *E. japonica* were the efficient species in retaining PM at different scales (i.e., leaf, individual tree, green area). The species like *Trifolium repens*, *Phyllostachys viridis*, were the least efficient plant species. The investigated species are all evergreen species, which will remove PM throughout the whole year, even in winter. So, we recommended that the plant species with the highest PM retention efficiency can be used in urban greening. Meanwhile, horticulture practices should also be considered to improve the leaf area index to improve their PM retention and air purification abilities.

## 1. Introduction

Atmospheric particulate matter (PM), defined as the sum of solid and liquid particles suspended in the air, is one of the fastest-growing types of environmental pollution in the world [1,2]. High levels of PM are considered very hazardous to human health, causing premature mortality, accelerated atherosclerosis, lung cancer, heart disease, and asthma [3,4,5]. PM can also cause other adverse impacts such as visibility, scattering and absorbing solar radiation, change cloud nucleation process, lead to photochemical smog, and exacerbate the greenhouse effect [6]. Thus, PM pollution is gaining wide attention around the world. Studies have shown that urban vegetation (e.g., leaves, tree barks) (e.g., [7,8,9,10]) can accumulate PM from the air more effectively than other building/land surfaces due to the extensive leaf area, the complex micromorphology, and the structure of the vegetation crown changes the turbulence of air movement above and within the canopies [11]. Therefore, mitigating and controlling PM pollution using urban forests has attracted more and more attention in recent years [12,13]. Given limited greening space in metropolitan areas, the most effective plant species removing PM should be selected for urban greening. Consequently, a quantitative assessment of the amount of PM retained by plant leaves at different levels (e.g., leaf, individual tree, green area) becomes an important issue.

At present, some studies have been carried out using a range of techniques, e.g., weighing method [14], membrane filter method [15], and the elution weighing method coupled with a particle size analysis [16], or microscopic images combining object-based image analysis [17], Image J software [18], or Image Pro-Plus [19]. Among the mentioned methods, the weighing method is often used to quantitatively estimate the PM retention amount on leaf surfaces. Collecting PM on leave surfaces completely is a prerequisite for using weighing methods. To collect PM deposited on leave surfaces, some researchers used water washing and then brushing to clean the sampled leaves [11,14]. However, the results of some studies (e.g., [20,21,22]) demonstrated that these cleaning methods could not completely elute the PM on the leaf surfaces. When an appropriate ultrasonic leaf cleaning procedure was added to the conventional cleaning method, the cleanliness of *Ginkgo biloba*, *Sophora japonica*, *Salix babylonica*, *Pinus tabuliformis*, and *Sabina chinensis* leaf surfaces could be improved markedly [23,24].

The components of PM in the atmosphere are complex, including insoluble mineral dust, highly water-soluble inorganic salts, and carbonaceous material. Water-soluble chemical compositions represent a great portion of the particle mass, which accounted for more than 1/3 of PM_2.5_ mass [25,26,27]. Organic compounds also constitute a major fraction of PM in urban areas, often over 30% of PM_2.5_ mass [28], most of which can be dissolved in chloroform [29]. However, the cleaning methods, as a single water washing [30], followed by scrubbing the leaves [14,31], can only separate and quantify the insoluble fraction deposited on leaf surfaces. The surfaces of leaves are covered with a cuticle that consists of cutin and waxes. Some particles can be trapped in epidermal wax [25]. So, some researchers used water washing, scrubbing leaves, followed by chloroform washing [11,15,32], and combining different pore membranes [15]. The mentioned method can only separate and quantify the insoluble fraction of the retained PM on leaf surfaces and trapped in wax. Therefore, it may underestimate the amount of particles on leave surfaces because water-soluble ions and chloroform dissolved organic compounds account for a big proportion of the total PM mass in some samples [25,28]. For the method of microscopic images analysis, it is difficult to count the number of PMs deposited on leaves with complex microstructures or aggregated particles. In addition, this method is time-consuming and only acquires a very limited area, limiting the amount of reliable data available for further analysis. 

Investigating the amount of water-soluble and chloroform dissolved organic compounds of PM and the small particles that can pass through the membrane pore (e.g., 0.1 µm, 0.2 µm) not only can help to determine the precise amounts of total PM retained on leaf surfaces but also help to understand the ability of leaves to get rid of the toxic pollutants through precipitation [33,34]. Therefore, few studies were undertaken to determine the water-soluble ions (Cl^−^, SO_4_^2−^, NO_3_^−^, NH_4_^+^, Ca^2+^, Na^+^, Mg^2+^, K^+^) using an ion chromatography [32]. However, they did not pay attention to the other water-soluble ions, small particles less than 0.1 or 0.2 µm, and the dissolved organic compounds. To achieve the total leaf surface PM, Hong et al. [35] used oven-dried washing elution liquids, but this method is time-consuming (maybe several days to reach constant weight) and did not consider the PM encapsulated in wax.

All inorganic and organic substances contained in a liquid in molecular, ionized, or microgranular suspended forms are defined as total dissolved solids (TDS), which can survive filtration through a filter/membrane with 2 µm pores. Thus, determining the TDS of the filter liquors pass through a membrane with <2 µm plus the insoluble particles intercepted by membranes with different pores (10 µm, 2.5 µm, 0.1 µm) provide a reasonable method to assess the total PM retained by leaves. In addition, there is poor comparability among the different research results when using the retained PM mass to assess the air purification ability of the urban trees [24] since the dust retention durations of the plants used in the research might be different. Thus, as an alternative, some researchers used the PM retention efficiency (the number of particles retained per unit leaf area per unit time) to assess the ability of the urban trees to retain PM (e.g., [24,31]).

In the present study, we used 20 plant species, all of which are widespread in temperate regions. We first washed some of the leaves in-situ using tap water and labeled them. Then we collected the labeled leaves after 7 d of exposure to heavy PM pollution weather in winter. We measured the surface PM (SPM), in-wax PM (WPM) and within different diameter classes using water and chloroform and brushing clean. The water-soluble (DPM) and organic-soluble (OPM) PM were investigated using a fast and time-saving method. The objectives of the present study were: (1) to compare and analyze the PM (including soluble and insoluble fractions) within different diameter classes retained on leaves of the investigated plant species based on different scales (i.e., leaf, individual tree, green area); (2) to estimate the PM retention efficiency of the investigated plant species at different scales. The results of our study could be useful when selecting greening plant species with high air purification abilities.

## 2. Results

### 2.1. Leaf Surface Micromorphology of Different Plant Species

**Figure 1:**
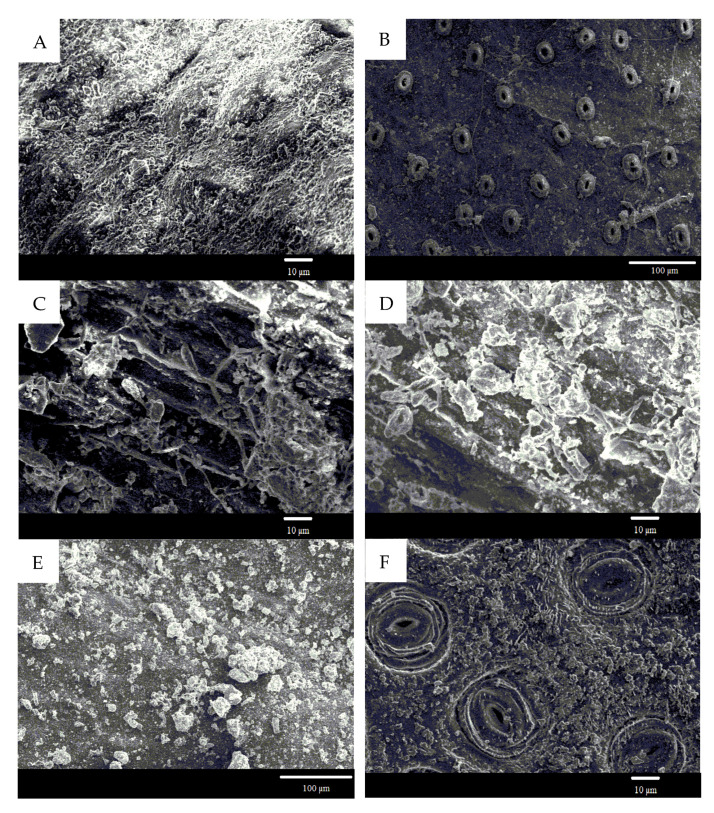

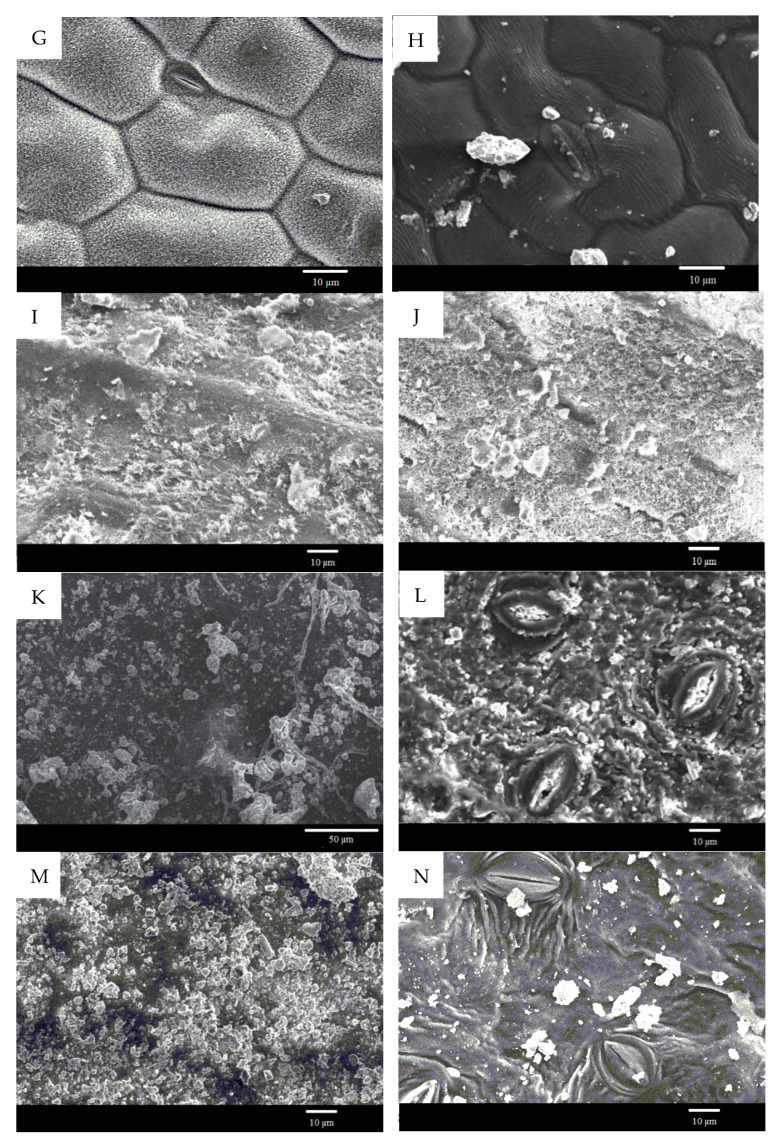
Scanning electron microscope pictures of *Buxus sinica* (**A**,**B**), *Pinus tabuliformis* (**C**,**D**), *Pittosporum tobira* (**E**,**F**), *Trifolium repens* (**G**,**H**), *Cedrus deodara* (**I**,**J**), *Ligustrum lucidum* (**K**,**L**) and *Viburnum odoratissinum* (**M**,**N**). Adaxial surface: (**A**,**C**,**E**,**G**,**I**,**K**,**M**); Abaxial surface: (**B**,**D**,**F**,**H**,**J**,**L**,**N**).

Figure 1 presents the leaf surface structural properties of the selected plant species. The adaxial surface of *Buxus sinica* was covered with convex cells. Plenty of particles were deposited on these cells or within the different-sized space among them (Figure 1A). The stomata (33 µm × 25 µm) was only distributed on the abaxial surface, with most small particles distributed around the stomatal apparatus (Figure 1B). There were some shallow furrows on the adaxial surface of *P. tabuliformis*, and abundant particles were retained within these furrows. The filamentous fungi on leaf surfaces seemed to form “bridges” between separated particles or aggregated particles (Figure 1C, D). The adaxial surfaces of *P. tobira* (Figure 1E), *Ligustrum lucidum* (Figure 1K), and *Viburnum odoratissinum* (Figure 1M) were relatively smooth and flat but retained many small particles. However, the abaxial surfaces of *P. tobira* (Figure 1F), *L. lucidum* (Figure 1L), and *V. odoratissinum* (Figure 1N) were more complexes than the adaxial surfaces. The platelet-shaped wax crystals were observed on the abaxial surface of *P. tobira* (Figure 1F). On the abaxial surfaces of *L. lucidum* (Figure 1L) and *V. odoratissinum* (Figure 1N), there were wide-spread furrows and ridges, with some particles retained on the ridges or within the furrows. However, a dense layer of wax crystals was observed on the adaxial surface of *T. repens* (Figure 1G). The wax crystals were also observed on both sides of *Cedrus deodara*, but some of them were fused (Figure 1I,J).

### 2.2. PM Elution Characteristics of Different Plant Species

**Figure 2:**
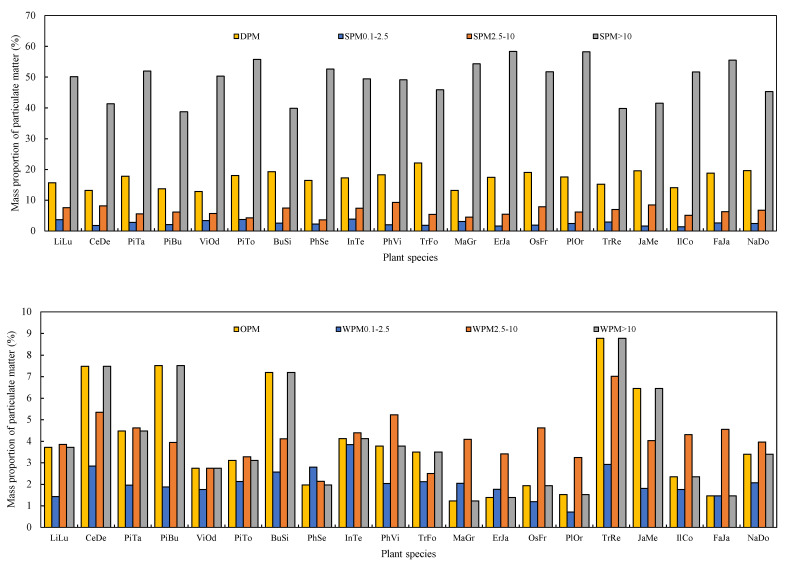
The mass proportions of eluted by water and brushing (SPM), chloroform and brushing (WPM) within different diameter classes (diameter > 10 µm, 2.5–10 µm, 0.1–2.5 µm), water-soluble (DPM), and organic-soluble (OPM) particulate matter retained on the investigated leaves.

Of the 20 species analyzed, the mass proportions of PM within the different diameter classes differed significantly among different species and different steps (Figure 2, ANOVA, *p* < 0.05). When the leaves were first cleaned with water and then brushed, the proportion of SPM, DPM, SPM_0.1–2.5_, SPM_2.5–10_, and SPM_>10_ ranged from 44.9% (*Pinus bungeana*) to 66.9% (*P. orientalis*), 12.9% (*V. odoratissimum*) to 22.1% (*Trachycarpus fortunei*), 1.4% (*Ilex cornuta*) to 3.8% (*Indocalamus tessellatus*), 3.6% (*Photinia serrulata*) to 9.3% (*P. viridis*), and 38.9% (*P. bungeana*) to 58.4% (*E. japonica*), respectively. 

Subsequently, when chloroform was applied to clean the leaves then brushing, a large proportion of PM was eluted from the leaf surfaces (Figure 2B). For the analyzed species, the proportions of WPM, OPM, WPM_0.1–2.5_, WPM_2.5–10_ and WPM_>10_ ranged from 14.1% (*P. orientalis*) to 31.7% (*P. bungeana*), 1.2% (*M. grandiflora*) to 8.8% (*T. repens*), 0.7% (*P. orientalis*) to 3.8% (*I. tessellatus*), 2.1% (*P. serrulata*) to 7.1% (*T. repens*), and 9.2% (*F. japonica*) to 25.9% (*P. bungeana*), respectively.

### 2.3. PM Retention Capacity of the Different Plant Species

**Figure 3:**
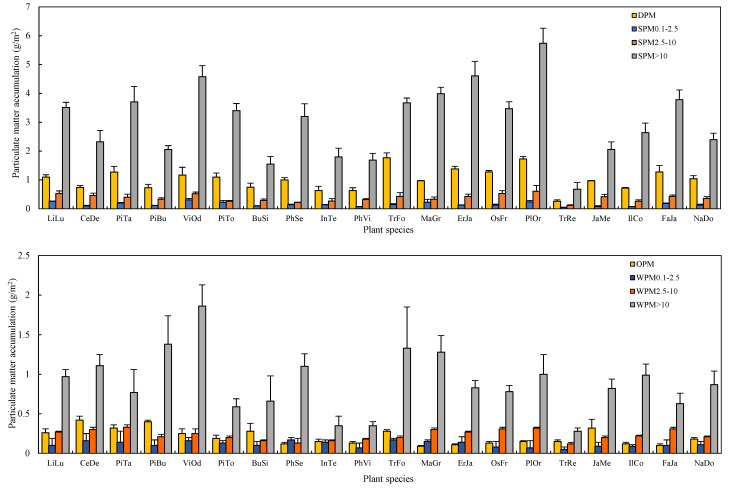
The mass of PM per unit leaf area eluted by water and brushing (SPM), chloroform and brushing (WPM) within different diameter classes (diameter > 10 µm, 2.5–10 µm, 0.1–2.5 µm), water-soluble (DPM), and organic-soluble (OPM) particulate matter retained on the investigated leaves.

**Figure 4:**
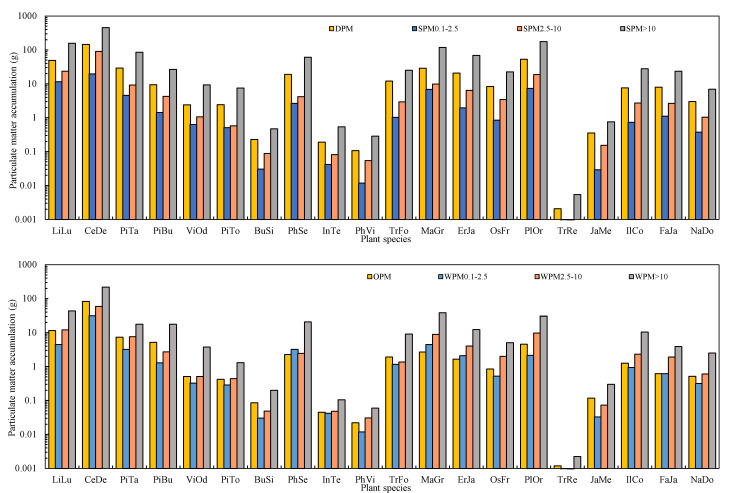
The mass of PM per tree eluted by water and brushing (SPM), chloroform and brushing (WPM) within different diameter classes (diameter > 10 µm, 2.5–10 µm, 0.1–2.5 µm), water-soluble (DPM), and organic-soluble (OPM) particulate matter retained on the investigated leaves.

**Figure 5:**
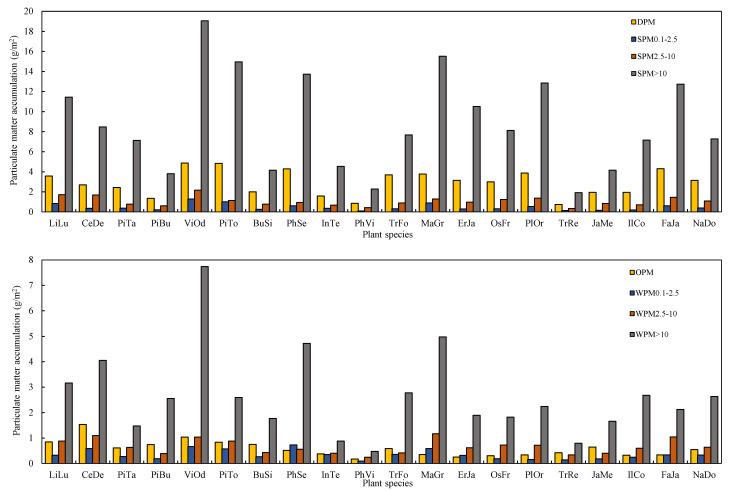
The mass of PM per unit green area eluted by water and brushing (SPM), chloroform and brushing (WPM) within different diameter classes (diameter > 10 µm, 2.5–10 µm, 0.1–2.5 µm), water-soluble (DPM), and organic-soluble (OPM) particulate matter retained on the investigated leaves.

The amounts of PM within different diameter classes captured by leaves based on per unit leaf area, per tree, and per unit green area all showed significant differences among species (Figure 3, ANOVA, *p* < 0.05). In general, the ranking presented in terms of capturing PM within different diameter classes was similar (Figure 3, Figure 4 and Figure 5). The species were divided into three groups (Figure 3). *P*. *orientalis*, *T. fortune, M. grandiflora, E. japonica, Osmanthus fragrans, F. japonica, V. odoratissimum, P. tabuliformis,* and *L. lucidum* showed the highest total PM retention (Figure 3). *T. repens* showed the lowest PM retention, while the remaining species showed intermediate PM retention capacity.

For the retained PM per tree, *C*. *deodara*, *P*. *orientalis*, *L. lucidum*, *M. grandiflora*, *P. tabuliformis* are the efficient plant species, but *T. repens*, *P*. *viridis*, *I. tessellatus*, *B. sinica* are the inefficient species due to the lower total leaf area of a tree. The remaining species showed intermediate PM accumulation (Figure 4).

As for the PM retention per unit green area, *V. odoratissimum*, *L. lucidum*, *M. grandiflora*, *P. tobira*, *C. deodara*, *P. serrulate*, *F. japonica*, *P*. *orientalis* showed the highest PM accumulation (Figure 5). *T. repens* is considerable the least efficient species for PM accumulation. At the same time, the remaining species showed intermediate PM accumulation. For the PM size fractions, the plant species always showed the same order as total PM.

### 2.4. The PM Retention Efficiency of the Different Tree Species

The plant species showed significant differences in *AE_leaf_*, *AE_plant_*, and *AE_land_* in various diameter classes and total PM (Table 1, ANOVA, *p* < 0.05). *AE_leaf_* of the different species varied between 22.7 (*T. repens*) and 169.8 (*P. orientalis*) mg/(m^2^·d). For DPM, PM_0.1–2.5_, PM_2.5–10_, PM_>10_, *AE_leaf_* ranged from 5.3 (*T. repens*) to 36.1 (*T. fortunei*), 1.0 (*T. repens*) to 6.3 (*V. odoratissimum*), 2.5 (*T. repens*) to 12.5 (*P. orientalis*), and 13.9 (*T. repens*) to 117.1 (*P. orientalis*) mg/(m^2^·d), respectively.

*AE_plant_* of SPM, DPM, PM_0.1–2.5_, PM_2.5–10_, PM_>10_ for different plant species varied from 0.2 to 14449.0, 0.1 to 2953.2, 0.1 to 399.2, 0.1 to 1836.1, 0.1 to 9260.5 mg/d, respectively. *AE_land_* of SPM, DPM, PM_0.1–2.5_, PM_2.5–10_, PM_>10_ for the different plant species varied from 64.1 to 558.6, 15.0 to 99.3, 1.9 to 26.3, 6.9 to 44.2, 39.3 to 388.8 mg/(m^2^·d), respectively.

In general, *P. orientalis*, *V. odoratissimum*, *P. tobira*, *P. serrulate*, *M. grandiflora*, and *F. japonica* were the most efficient plant species in retaining SPM and its diameter classes, and the species *T. repens* and *P. viridis* were the least efficient plant species in removing atmospheric particles.

### 2.5. Relationships between Leaf PM Retention and Surface Wettability, Surface Free Energy

The plant species showed significant differences in leaf contact angle (CA) and surface free energy (SFE) among the plant species (Table 2, ANOVA, *p* < 0.05). Strong negative correlations were observed between CA on adaxial surfaces and PM within the different diameter classes (*p* < 0.05), but positive correlations between SFE, polar and dispersive components within the different diameter classes were observed (*p* < 0.05).

## 3. Discussion

### 3.1. Methods for Quantifying PM on Leave Surfaces

Completely collecting the PM retained on leaf surfaces is the basis to accurately evaluate PM retention ability. The present results showed that only 44.9%−66.9% of the PM retained on leaves were eluted by water and brush cleaned, indicating that the conventional washing method cannot accurately assess the PM retention capacity of plants [8,24,25]. On average, a removal rate of 29–46% was observed by Liu et al. [24] using the water and brush cleaning method. In a study conducted by Wang et al. [14], it was observed that 31.9 mm precipitation under natural conditions could wash off 50% and 62% of the PM on leaves of *L. lucidum* and *V. odoratissimum*, respectively. However, it had no obvious influence on eluting the PM on *P. tabuliformis* leaves. An investigation carried out by Xu et al. [22] found that 51−70% of PM could be removed from the leaves of *E. japonicus*, *A. altissima*, *S. japonica*, *P. tomentosa*. Simulated rainfall of 20 mm can remove 30–40% of PM from Scots pine [36]. The TSP, PM_10–100_, PM_2.5–10_, and PM_2.5_ removal rate of 49.3–87.1%, 22.8–71.5%, 40.4–92.0%, and 50.7–91.3% was found by Zhang et al. [21]. As a consequence, the effects of rainfall on removing PM retained by leave surfaces were dependent on plant species, leaf surface microstructure, the growth conditions, rainfall intensity, duration, and volume [14,22]. For some plant species, the rainfall under natural conditions had fewer effect on removing PM retained on leave surfaces. However, it does not mean that the plants have fewer effects on PM removal because there is probably a dynamic trend in PM deposition on leave surfaces. Washing off PM from leaves can occur during precipitation, and then new deposition, meaning the accumulation of pollutants will continue more or less throughout the whole season, which depends on plant species and weather conditions.

To overcome the shortage of water and brush cleaning, some researchers used water and chloroform (often used to extract cuticular wax of plant leaves) washing successively, which was based on the structure and chemical composition of leaf cuticular wax can trap particles. Popek et al. [37] used chloroform to elute the in-wax PM. They found that in-wax PM contributed about 40% to total PM. In a study took in Beijing, Xu et al. [32] found that 35% of particles were trapped in wax. The particles trapped in wax are immobilized and phytostabilised in epidermal wax and thus lowers the negative impact on human health to a greater degree than the SPM. On the other hand, in-wax particles are very difficult to remove by rainfall or wind, so there will be no new deposition when saturation.

The high proportion of both DPM (12.9%−22.1%) and OPM (1.2%−8.8%) indicates that DPM and OPM are also important components of total PM deposited on leave surfaces. However, many previous studies did not consider the soluble components, meaning that the previous methods might underestimate plants’ PM retention ability to a large degree, especially for the soluble particles. Hence, it is recommended that the proper methods should be used to quantify the water-soluble and chloroform-soluble particles. In the present study, we used the TDS to determine the water-soluble particles, which is a fast, easy-to-operate and cost-saving method.

### 3.2. The Effects of Leaf Surface Microstructures on PM Retention

Urban plants accumulate PM in different ways, at least partly depending on the leaf morphologic structures (e.g., leaf wax, leaf wettability, and stomatal density and leaf area) (e.g., [38,39]). In the present study, different plant species showed obvious differences in leaf microstructure, resulting in great differences in PM retention capacity and efficiency among the investigated plant species. The leaves of *T. repens* are covered with dense wax crystals but with the lowest capacity and efficiency in PM retention, indicating that the leaves with wax crystals would have a lower capacity for capturing PM. *V. odoratissimum*, a species that retained a high level of particles and also had a high efficiency in retaining small particles. These results indicated that the leaves with the microstructure of furrows and ridges would have a high capacity for capturing PM. From the SEM images, we can observe that particles, especially the smaller ones could deposit within the furrows. There were also furrows on sticky leaves of *P*. *tabuliformis*; the fungi on such surfaces can act as bridges to combine the small particles, forming a bigger one. Consequently, *P*. *tabuliformis* exhibited a higher retention efficiency for the bigger diameter class.

As for the effects of leaf wettability on PM retention, negative correlations were found between leaf CA on adaxial surfaces and PM retention amounts in the present study. The leaves of *T. repens* are highly non-wettable (with a CA of 131.2 ± 4.3°) showed the lowest PM retention ability; a particle on such surfaces is like a fakir on his bed of nails [39]. On such surfaces, the contact area between a particle and the underlying surface is reduced due to microroughness caused by wax crystals. Thus, the particles can be washed out easily from such a surface by rain, fog, or dew (Lotus effect). However, for wettable leaves with small CA, the contact area between pollutants (e.g., particles) and the leave surfaces is large. As a consequence, the retained PM does not easily fall off the leave surface; the ability of PM retention will be higher than that of non-wettable leaves.

In terms of the effects of leaf SFE on PM retention, positive correlations were found in the present study. SFE and its components are physico-chemical properties of all materials [40]. Different plant leaves have different chemical compositions, affecting the SFE and its components. In the present study, we found that SFE is mainly contributed by dispersive components, and which is close related with leaf chemical composition, i.e., hydroxyl fatty acid, aliphatic compounds, and nonpolar or weak polar material such as cyclic compounds. In such conditions, when the ambient particles moved close enough to the leaf surfaces, the particles were adsorbed under the effect of dispersive forces. Thus, the greater the dispersive component of the SFE, the stronger the adsorption effect of particles on leave surfaces. Therefore, the leaf PM retention amounts and dispersive components were positively correlated. The concentration of the polar components to SFE is relatively small, which may be lead to smaller effects on PM retention. However, PM composition is very complicated, the influence of the interaction between functional groups of leave surfaces and PM can not be ignored. These results imply that the degree to which amount of PM retention by leaves was determined by the SFE and its components. In this regard, the estimation of SFE constitutes an easy and valuable way to quantify the amount of PM deposition for a particular plant surface.

### 3.3. The Proper Index for Evaluating the PM Accumulation of Leave Surfaces of Plant Species

The differences in PM accumulation and its size fractions among species could be used for urban greening plant species selection during urban or suburb afforestation. The amount of PM retained per unit leaf area is often used to assess the PM retention abilities of the plants at present [31,32,39]. However, this index has some shortcomings in comparing the results of different studies due to the possible difference in dust retention duration, different seasons, different environmental conditions, and different leaf stages [11,14,39]. In addition, a larger amount of PM on leaves does not mean that the PM removal efficiency will be higher. Therefore, as an alternative, some researchers used the retention efficiency (the number of particles retained on a unit leaf area per unit time) to assess the PM retention abilities of the plants [31]. However, these studies only use the unit leaf area scale.

The order in leaf PM retention efficiencies within the different diameter classes (i.e., PM_>10_, PM_2.5–10_, PM_0.1–2.5_) based on per leaf, per plant, and per unit green area showed significant differences among species (Table 1). These results suggest that a plant species with higher PM retention efficiency per unit leaf area would not necessarily have higher air purification ability on the individual tree scale or uint green area scale since these depends on the total leaf area/LAI of a tree. Zuo et al. [41] found that the diameter at breast height (DBH) of *P. bungeana* had significant effects on PM retention amount on a single tree which might be because DBH had effects on crown radius and LAI; thus the total leaf area. Furthermore, the tree size and health condition will be varied in different environmental conditions; the LAI will also change. It is recommended that the PM retention efficiency per unit green area (the product of PM retention efficiency per unit leaf area and LAI) may be suitable to be an index of PM retention by plants since this index can be a signal of the efficiency of the per-unit green area to accumulation of PM.

Besides considering a city-scale tree cover, it is timely to examine the health impacts at PM “hotspot” (e.g., near busy roads, highways) and/or where some of the most vulnerable population groups (e.g., young children, elderly people, the people with pre-existing lung/heart disease). For mitigation and control of PM pollution, it is important to select plant species according to the PM retention efficiency, the characteristics of air pollution, and local climate. If considering the PM retaining efficiency per unit leaf area, *P. orientalis*, *F. japonica*, *L. lucidum*, *P*. *tobira*, *V. odoratissimum* is recommended because the higher PM retaining efficiency and the deposited PM is easy to renew due to rainfall. If considering the PM retaining efficiency per unit green area, *V. odoratissimum*, *M. grandiflora*, *P. serrulate*, *P. tobira*, *L. lucidum*, *P. orientalis*, *F. japonica*, *C. deodara* is recommended. Meanwhile, horticulture practices should be considered to improve the LAI of the plant species to greatly improve their particle retention abilities on both the single tree and stand levels, even at the city scale, for improving the air quality.

## 4. Materials and Methods

### 4.1. Plant Materials and Leaf Sampling

Twenty plant species, widespread in temperate regions, were selected for this study (Table 3) in Xi’an University of Architecture & Technology (34°24′53″ N, 108°96′26″ E, and elevation 421 m), Xi’an, China. The distances between sampling plant species and the nearly main road (Yanta Road) were about 50 m. Thus, the environmental conditions of these sampling plants were similar. For each plant species, two to four individual plants depending on their occurrence on the campus under good growth conditions were selected except *T. repens*. We first washed the leaves of the selected plants using tap water and labelled them on 18 December 2018. Then, the labeled leaves with small branches were collected after seven days of exposure. The small branches bearing leaves were placed in labeled ziplock bags (15#, 400 mm × 700 mm), transported to the laboratory, and kept in a 4 ºC fridge until analysis.

### 4.2. Experimental Methods

#### 4.2.1. Measurement of Leaf PM Retention Amount

Three batches of leaves were initially prepared for each species for determining the PM retention amount. For each batch, 8−10 pieces for big leaves, 30−40 pieces for small leaves, or 50−80 bunches for needles were selected. The leaves were cleaned as the following steps, 

(1) Water cleaning: Every batch of leaves was placed in glass containers with 300 mL of deionized water and stirred for 60 s with a glass rod. After this, the leaves were scrubbed by a no-hair-loss brush and washed with 50 ml of deionizing water. These represent particles that can be washed off the leaves during rainfall and labelled as SPM.

(2) Chloroform cleaning: Each sample of leaves after step (1) was washed with 150 ml of chloroform for 15 s. These represent particles that were trapped in wax and labeled as WPM. 

(3) Filtration: Each suspension after steps (1) and (2) was pumped through three types of microporous membranes with the pore size of 10, 2.5, and 0.1 µm successively. Here, we obtained three fractions of PM: (i) PM_>10_ (particles intercepted by the membrane with pore size 10 µm, labeled as PM_>10_), (ii) PM_2.5‒10_ (particles intercepted by the membrane with pore size 2.5 µm, labeled as PM_2.5–10_), and (iii) PM_0.1–2.5_ (particles intercepted by the membrane with pore size 0.1 µm, labeled as PM_0.1–2.5_). For the filtration procedure, all the membranes used for analysis were first soaked in deionized water for 2 h and then dried at 105 ºC in a drying chamber for 6–8 h to remove soluble impurities. The filters were then put in a balancing chamber for 48 h to stabilized the humidity change. Every filter was weighed before (*M*_1_) filtration three times to reduce the potential errors using FA2004 balance (Shanghai Precision Scientific Instrument Co. Ltd, Shanghai, China). Every loaded filter was subsequently dried for about 24 h in a drying chamber at 40 °C, and then re-weighed (*M*_2_) as above. The mass of PM_>10_, PM_2.5–10_, and PM_0.1–2.5_ deposited on leaves were calculated as (*M*_2_−*M*_1_).

(4) Measurements of the TDS of elution passing through the membrane with a pore size of 0.1 µm after step (3). The TDS (*C*, mg/L) was measured using a conductivity meter (Leici DDS-307A, INESA Analytical Instrument Co. Ltd, Shanghai, China). The mass of PM dissolved in water (DPM) and chloroform (OPM) was calculated as *C* multiplying the volume of the filter liquor (*V*, L).

#### 4.2.2. Measurement of Plant Characteristics

##### Measurement of Leaf Area

The surface area for broad-leaved species for every batch of leaves was analyzed using an automatic image analysis software Image J (v.1.51j8, Wayne Rasband, National Institutes of Health, Bethesda, Maryland, USA) after scanning (HP Scanjet G2410, HP Inc., Palo Alto, California, USA). The leaf area of conifer needles was determined based on Equation (1).

(1) $$A=2L(1+\frac{\pi }{n})\sqrt{\frac{nV^{\prime }}{\pi L}}$$

where *L*, *n*, and *V′* are the average length, number, and volume of conifer needles, respectively.

##### Measurements of Above-Ground Growth Status

LAI of each plant species was measured based on the method described by Chen et al. [42]. The crown diameter was measured using a tape in the direction of west-east and south-north, and the geometric mean value was calculated as the crown diameter. Tree height was measured using the trigonometric leveling method [43]. Diameters at breast height (DBH) or ground level were measured at the height of 1.3 m or 0.1 m above ground using a tape for trees and shrubs, respectively.

#### 4.2.3. Observation of Leaf Surface Microstructure

For every species, three pieces (about 5 mm × 5 mm for broad-leaved species, and 5 mm in length for conifer needles) were cut from the center of the leaves were attached to the aluminum stub with double-sided adhesive tape, sputter-coated with gold for 30 s at a current of 10 mA (JFC-1600, JEOL, Akishima, Tokyo, Japan) and examined with JSM-6510LV scanning electronic microscopy (working conditions: vacuum, resolution: 3 nm, JEOL, Akishima, Tokyo, Japan) at an accelerating voltage of 10 kV. The microstructure of the adaxial and abaxial surfaces was observed at ten randomly selected positions on the leaves.

#### 4.2.4. Measurements of Leave Contact Angle

Contact angles (CA, *θ*) were determined on adaxial surfaces using distilled water and diiodomethane (purity ≥ 99%, Beijing Chemical Reagent Factory, Beijing, China) at room temperature using an optical contact angle meter (Kino SL200A, KINO Industry Co., LTD, Somerville, Boston, Massachusetts, USA). For every species, fifteen pieces (about 5 mm × 5 mm for broad-leaved species, and 5 mm in length for conifer needles) were cut from the center of the leaves discarding the midvein were mounted on a microscope slide with double-sided adhesive tape. Droplet volumes of 6 µL or 2 µL of distilled water and 2 µL of diiodomethane were selected based on the unit leaf area, the properties of the liquids, and the effect of droplet volume on the contact angle (i.e., contact angles were independent of the droplet volume for volumes between 1 and 10 µL [44].

#### 4.2.5. Calculation of Leaf SFE

The solid SFE was determined by CA measurements using a set of liquids with different surface free energies [45] based on the theory of Young [46]:

*γ*_sl_ = *γ*_s_ − *γ*_l_cos*θ* (2)

where *γ*_s_ and *γ*_l_ are the SFE of the solid and liquid (mJ/m^2^), respectively; *γ*_sl_ is the solid/liquid interfacial energy, *θ* is the CA (*θ*).

According to Fowkes’ theory, the SFE of a solid could be divided into two components [47]:

*γ* = *γ*^d^ + *γ*^p^ (3)

where *γ*^d^ and *γ*^p^ are the dispersive and polar components (mJ/m^2^), respectively.

Owens–Wendt [48] developed Fowke’s theory and established Equation (4):

(4) $$\gamma_{s}{}_{l}=\gamma_{l}+\gamma_{s}-2\sqrt{\gamma_{s}{}^{d}\gamma_{l}{}^{d}}-2\sqrt{\gamma_{s}{}^{p}\gamma_{l}{}^{p}}$$

where *γ*_s_^d^ and *γ*_l_^d^ are the dispersive components of SFE of solid and liquid (mJ/m^2^), respectively; *γ*_s_^p^ and *γ*_l_^p^ are the polar component of SFE of solid and liquid (mJ/m^2^), respectively.

Combining Equation (4) and (2) yields:

(5) $$\gamma_{l}(1+\cos\theta )=2(\sqrt{\gamma_{l}{}^{p}\gamma_{s}{}^{p}}+\sqrt{\gamma_{l}{}^{d}\gamma_{s}{}^{d}})$$

For all the surfaces evaluated, the SFE and its components, i.e., the dispersive and polar components were calculated, considering the *γ*, *γ*^p^, and *γ*^d^ are 72.8, 51.0, and 21.8 mJ/m^2^ (distilled water) and 50.8, 2.3, and 48.5 mJ/m^2^ (diiodomethane) [49].

### 4.3. Data Presentation

The proportion of the different-sized particles eluted by each cleaning step was calculated by Equation (6):

(6) $$P_{i,j}=\frac{M_{i,j}}{\sum M_{i,j}}.\;$$

where *P_i,j_* and *M_i,j_* are the mass proportion (%) and the mass (g) of the particles within the *j* diameter class eluted by the cleaning step *i* from the leaf surfaces, respectively.

The WPM can not be removed by rainfall or wind, so only the retention efficiency of SPM of different plant species was considered in the present study. The retention efficiency of SPM on a unit leaf surface area (*AE_leaf_*) was calculated by using Equation (7):

(7) $${\mathrm{AE}}_{\mathrm{leaf}}=\frac{M_{j}}{t}$$

where *M_j_* is the mass of the particles within the *j* diameter class retained on a unit of leaf area (g/m^2^); *t* is the time of exposure (7 days).

The SPM retention efficiency of an individual tree (*AE_plant_*) of different plant species was calculated by using Equation (8):

*AE_plant_* = *AE_leaf_* × *LA* (8)

where *LA* is the total leaf area of an individual tree. *LA* can be calculated by using Equation (8):

*LA* = *LAI* × *D*^2^ × π/4 (9)

where *LAI* is the leaf area index (m^2^/m^2^); *D* is the crown diameter (m).

The SPM retention efficiency on a unit greening land (*AE_land_*) of different plant species was calculated by using Equation (10):

*AE_land_* = *AE_leaf_* × *LAI* (10)

### 4.4. Data Analysis

One-way analysis of variance (ANOVA) was undertaken using Minitab (v.17.1.0, Minitab Inc., State College, Pennsylvania, USA) statistical packages to estimate the differences in the retention capacities of the different sized particles, SPM, WPM, total PM, and mass proportion of PM among the 20 species. When ANOVA indicated significant differences among species, the pairs of species that exhibited significant differences were determined using Tukey’s multiple means comparison tests. The relationships between variables were assessed with regression procedures. A given effect was assumed significant at *p* < 0.05. K-means clustering was used to group the species into three sets with low, intermediate, or high ability to accumulate PM. Clustering was run for the three PM size fractions, combining surface, wax-bound particles of each fraction and soluble fractions. 

## 5. Conclusions

Measurements of the water-soluble and in-wax PM are the key to accurately quantify the PM retention capacity and efficiency of plants because they accounted for a large proportion of the accumulated PM by plant leaves, 12.9–22.1%, 14.1–31.7% of the total PM. Plant species showed significant differences in PM retention capacity and efficiency by leaves based on per unit leaf area, per plant, and per unit green area. The ranking presented in terms of capturing PM within different diameter classes can be used to select species for atmospheric PM pollution removal in PM pollution regions. Even though the mass of PM retained on a unit leaf area is the most commonly used index to assess the retention abilities of the plants. It may be incomparable with other results. Therefore, PM retention efficiency is an alternative index to assess the PM retention abilities of plant species. Since the quantity of PM within different diameter classes captured by plant leaves depends on the PM retention amount per unit leaf area and leaf area index, efficient plant species and plant configuration designs considering different life forms and leaf habits can be used to decrease human exposure to the pollutants.

## Figures and Tables

**Table 1:** The retention efficiency (AE) of the investigated plant species in retaining surface PM (SPM) within different diameter classes (particle diameter > 10 µm, 2.5–10 µm, 0.1–2.5 µm) and water-soluble PM (DPM).

Species	AE*_leaf_* (mg/m^2^·d)					AE*_plant_* (mg/d)					AE*_land_* (mg/m^2^·d)				
	Diameter Class					Diameter Class					Diameter Class				
	DPM	PM_0.1–2.5_	PM_2.5–10_	PM_>10_	SPM	DPM	PM_0.1–2.5_	PM_2.5–10_	PM_>10_	SPM	DPM	PM_0.1–2.5_	PM_2.5–10_	PM_>10_	SPM
*LiLu*	22.5	5.3	10.8	71.6	110.2	999.5	236.2	481.6	3189.3	4906.6	73.2	17.3	35.3	233.5	359.3
*CeDe*	15.1	2.0	9.4	47.4	73.9	2953.2	399.2	1836.1	9260.5	14,449.0	55.1	7.5	34.3	172.8	269.7
*PiTa*	25.9	4.1	8.2	74.7	112.9	594.5	93.6	187.2	1736.6	2611.9	49.8	7.8	15.7	145.4	218.7
*PiBu*	14.9	2.2	6.7	42.0	65.8	192.2	29.0	86.9	542.5	850.6	27.6	4.2	12.5	77.8	122.1
*ViOd*	23.9	6.3	10.6	93.5	134.3	48.7	12.9	21.6	190.6	273.8	99.3	26.3	44.2	388.8	558.6
*PiTo*	22.5	4.7	5.3	69.4	101.9	49.7	12.9	21.6	190.6	274.8	98.8	20.7	23.4	305.3	448.2
*BuSi*	15.3	2.0	5.9	31.6	54.8	4.7	0.6	1.8	9.6	16.7	41.0	5.5	15.9	84.8	147.2
*PhSe*	20.4	2.9	4.5	65.3	93.1	386.2	54.1	85.0	1235.9	1761.2	87.6	12.3	19.3	280.2	399.4
*InTe*	12.9	2.9	5.5	36.7	58.0	3.9	0.9	1.7	11.1	17.6	32.4	7.2	13.9	92.6	146.1
*PhSu*	12.9	1.4	6.5	34.5	55.3	2.2	0.2	1.1	5.9	9.4	17.4	1.9	8.8	46.6	74.7
*TrFo*	36.1	3.1	8.8	74.9	122.9	246.8	20.9	60.0	511.6	839.3	75.5	6.4	18.3	156.5	256.7
*MaGr*	19.8	4.7	6.7	81.4	112.6	588.7	139.6	200.3	2421.7	3350.3	77.0	18.3	26.2	316.8	438.3
*ErJa*	28.2	2.7	8.8	94.1	133.8	421.2	38.7	131.3	1407.1	1998.3	64.2	6.1	20.0	214.5	304.8
*OsFr*	26.1	2.7	10.8	70.8	110.4	169.7	17.2	70.3	460.0	717.2	61.1	6.2	25.3	165.7	258.3
*PlOr*	35.3	4.9	12.5	117.1	169.8	1080.1	149.8	380.8	3583.7	5194.4	79.1	11.0	27.9	262.4	380.4
*Tr* *Re*	5.3	1.0	2.5	13.9	22.7	0.1	0.1	0.1	0.1	0.4	15.0	2.9	6.9	39.3	64.1
*JaMe*	19.8	1.6	8.6	42.0	72.0	7.2	0.6	3.1	15.4	26.3	40.0	3.3	17.3	84.9	145.5
*IlCo*	14.7	1.4	5.3	53.9	75.3	154.1	15.0	55.7	565.2	790.0	39.8	3.9	14.4	146.0	204.1
*FaJa*	26.1	3.7	8.8	77.1	115.7	161.9	22.8	54.4	478.0	717.1	88.0	12.4	29.6	260.0	390.0
*NaDo*	21.2	2.7	7.3	49.0	80.2	61.1	7.6	21.2	141.0	230.9	64.3	8.0	22.3	148.4	243.0

**Table 2:** Leaf surface wettability and surface free energy of plant species.

Species	Contact Angle (°)		Surface Free Energy (mJ/m^2^)		
	Water	Diiodomethane	*γ* ^p^	*γ* ^d^	*γ*
*LiLu*	76.3 ± 8.8	55.9 ± 1.7	9.1	25.4	34.5
*CeDe*	68.8 ± 9.7	58.7 ± 6.3	15.0	22.0	37.0
*PiTa*	84.3 ± 4.6	54.9 ± 3.9	4.6	27.8	32.4
*PiBu*	73.3 ± 8.5	62.1 ± 7.4	12.8	20.8	33.6
*ViOd*	73.5 ± 7.3	55.2 ± 5.6	10.7	25.2	35.9
*PiTo*	80.4 ± 4.6	62.9 ± 3.7	8.3	21.9	30.2
*BuSi*	83.4 ± 9.4	54.9 ± 5.8	5.0	27.6	32.6
*PhSe*	63.3 ± 8.6	49.5 ± 8.7	16.2	26.4	42.6
*InTe*	87.5 ± 10.4	58.7 ± 3.5	3.9	26.1	30.0
*PhSu*	83.4 ± 7.2	56.5 ± 4.4	5.3	26.7	32.0
*TrFo*	59.7 ± 10.3	55.1 ± 5.9	20.9	22.5	43.4
*MaGr*	68.2 ± 14.2	54.4 ± 3.7	14.1	24.5	38.6
*ErJa*	49.7 ± 15.4	50.1 ± 8.5	27.3	23.5	50.8
*OsFr*	71.4 ± 6.0	51.1 ± 3.7	11.0	27.2	38.2
*PlOr*	66.3 ± 10.5	46.3 ± 6.9	13.2	28.9	42.1
*TrRe*	131.2 ± 4.3	104.9 ± 4.4	0.1	7.3	7.4
*JaMe*	80.1 ± 7.4	63.5 ± 6.1	8.7	21.5	30.2
*IlCo*	90.6 ± 10.1	59.9 ± 9.4	2.9	26.1	29.0
*FaJa*	68.7 ± 6.9	51.7 ± 4.7	12.9	26.3	39.2
*NaDo*	73.2 ± 4.5	55.9 ± 5.3	11.0	24.7	35.7

**Table 3:** Families, life forms, leaf shapes, and texture of the investigated plant species. The results from the clustering analysis with surface and wax dissolved fractions of PM_>10_, PM_2.5–10_, PM_0.1–2.5_, water-soluble and organic-soluble PM and total PM as variables. Cluster 1 had the smallest quantity of deposited PM, and cluster 3 had the largest.

Species	Abbreviation	Families	Life Form	Leaf Shape	Texture	Cluster
*Ligustrum lucidum*	*LiLu*	Oleaceae	Tree	Ovate, long ovate or elliptic to broadly elliptic	Leathery	3
*Cedrus deodara*	*CeDe*	Pinaceae	Tree	Needle	Leathery	3
*Pinus tabuliformis*	*PiTa*	Pinaceae	Tree	Needle	Leathery	1
*Pinus bungeana*	*PiBu*	Pinaceae	Tree	Needle	Leathery	1
*Viburnum odoratissimum*	*ViOd*	Caprifoliaceae	Shrub	Elliptic, rectangular-elliptic to obovate	Leathery	3
*Pittosporum tobira*	*PiTo*	Pittosporaceae	Shrub	Obovate or obovate lanceolate	Leathery	3
*Buxus sinica*	*BuSi*	Buxaceae	Shrub	Obovate to oblong ovate	Leathery	1
*Photinia serrulata*	*PhSe*	Rosaceae	Shrub	Obovate or obovate ellipse	Leathery	3
*Indocalamus tessellatus*	*InTe*	Gramineae	Herb	Elliptic lanceolate	Papery	1
*Phyllostachys viridis*	*PhVi*	Gramineae	Herb	Oblong lanceolate or lanceolate	Papery	1
*Trachycarpus fortunei*	*TrFo*	Palmae	Tree-dwelling	3/4 orbicular or suborbicular	Leathery	2
*Magnolia grandiflora*	*MaGr*	Magnoliaceae	Tree	Ellipse, oblong ellipse or obovate ellipse	Leathery	3
*Eriobotrya japonica*	*ErJa*	Rosaceae	Tree	Lanceolate, oblanceolate, obovate, or oblong	Leathery	2
*Osmanthus fragrans*	*OsFr*	Oleaceae	Tree	Elliptic, oblong or elliptic lanceolate	Leathery	2
*Platycladus orientalis*	*PlOr*	Cupressaceae	Tree	Squamiform	Leathery	2
*Trifolium repens*	*TrRe*	Leguminosae	Herb	Ternate palmate leaf	Papery	1
*Jasminum mesnyi*	*JaMe*	Oleaceae	Shrub	Compound	Leathery	2
*Ilex cornuta*	*IlCo*	Aquifoliaceae	Shrub	Quadrangular oblong or ovate	Leathery	2
*Fatsia japonica*	*FaJa*	Araliaceae	Shrub	Palmate lobed	Leathery	2
*Nandina domestica*	*NaDo*	Berberidaceae	Shrub	Elliptic or elliptic lanceolate	Leathery	2

## Data Availability

Not applicable.

## References

[1] Dominick D., Juahir H., Latif M.T., Zain S.M., Aris A.Z. (2012). Spatial assessment of air quality patterns in Malaysia using multivariate analysis. Atmos. Environ..

[2] Lim C.H., Ryu J., Choi Y., Joen S.W., Lee W.K. (2020). Understanding global PM_2.5_ concentrations and their drivers in recent decades (1998–2016). Environ. Int..

[3] World Health Organization (WHO) (2017). Evolution of WHO Air Quality Guidelines: Past, Present and Future.

[4] Wang H.X., Maher B.A., Ahmed I.A.M., Davison B. (2019). Efficient removal of ultrafine particles from diesel exhaust by selected tree species: Implications for roadside planting for improving the quality of urban air. Environ. Sci. Technol..

[5] WHO Health Effects of Particulate Matter: Policy Implications for Countries in Eastern Europe, Caucasus and Central Asia. http://www.euro.who.int/__data/assets/pdf_file/0006/189051/Health-effects-of-particulate-matter-final-Eng.pdf.

[6] Li L., Wang W., Feng J.L., Zhang D.P., Li H.J., Gu Z.P., Wang B.J., Sheng G.Y., Fu J.M. (2010). Composition, source, mass closure of PM_2.5_ aerosols for four forests in eastern China. J. Environ. Sci..

[7] Barwise Y., Kumar P. (2020). Designing vegetation barriers for urban air pollution abatement: A practical review for appropriate plant species selection. Clim. Atmos. Sci..

[8] Tong Z.M., Baldauf R.W., Isakov V., Deshmukh P., Zhang K.M. (2016). Roadside vegetation barrier designs to mitigate near-road air pollution impacts. Sci. Total Environ..

[9] Weerakkody U., Dover J.W., Mitchell P., Reiling K. (2018). Evaluating the impact of individual leaf traits on atmospheric particulate matter accumulation using natural and synthetic leaves. Urban For. Urban Gree..

[10] Flett L., McLeod C.L., McCarty J.L., Shaulis B.J., Fain J.J., Krekeler M.P.S. (2021). Monitoring uranium mine pollution on Native American lands: Insights from tree bark particulate matter on the Spokane Reservation, Washington, USA. Environ. Res..

[11] Sæbø A., Popek R., Nawrot B., Hanslin H.M., Gawrońska H., Gawronski S.W. (2012). Plant species differences in particulate matter accumulation on leaf surfaces. Sci. Total Environ..

[12] Abhijith K.V., Kumar P. (2020). Quantifying particulate matter reduction and their deposition on the leaves of green infrastructure. Environ. Pollut..

[13] Nowak D.J., Hirabayashi S., Doyle M., McGovern M., Pasher J. (2018). Air pollution removal by urban forests in Canada and its effect on air quality and human health. Urban For. Urban Green..

[14] Wang H.X., Shi H., Wang Y.H. (2015). Dynamics of the captured quantity of particulate matter by plant leaves under typical weather conditions. Acta Ecol. Sin..

[15] Dzierzanowski K., Popek R., Gawrońska H., Sæbø A., Gawroński S.W. (2011). Deposition of particulate matter of different size fractions on leaf surfaces and in waxes of urban forest species. Int. J. Phytoremediat..

[16] Zhang Z.D., Xi B.Y., Cao Z.G., Jia L.M. (2014). Exploration of a quantitative methodology to characterize the retention of PM_2.5_ and other atmospheric particulate matter by plant leaves: Taking *Populus tomentosa* as an example. Chin. J. Appl. Ecol..

[17] Yan J.L., Lin L., Zhou W.Q., Ma K.M., Pickett S.T.A. (2016). A novel approach for quantifying particulate matter distribution on leaf surface by combining SEM and object-based image analysis. Remote Sens. Environ..

[18] Sternberg T., Viles H., Cathersides A., Edwards M. (2010). Dust particulate absorption by ivy (*Hedera helix* L) on historic walls in urban environments. Sci. Total Environ..

[19] Li Y.F., Yang G.L., Li F.G., Yu L.S. (2005). Processing and analysis for micrograph of PM_2.5_ in atmosphere. Opto-Electron. Eng..

[20] Wang Z.H., Li J.B. (2006). Capacity of dust uptake by leaf surface of Euonymus japonicus Thunb. and the morphology of captured particle in air polluted city. Ecol. Environ..

[21] Zhang L., Zhang Z.Q., Chen L.X., McNulty S. (2019). An investigation on the leaf accumulation-removal efficiency of atmospheric particulate matter for five urban plant species under different rainfall regimes. Atmos. Environ..

[22] Xu X.W., Zhang Z.M., Bao L., Li M., Yu X.X., Fan D.X., Lun X.X. (2017). Influence of rainfall duration and intensity on particulate matter removal from plant leaves. Sci. Total Environ..

[23] Liu H.H., Cao Z.G., Jia L.M., Li X.Z., Hao L.F., Liu J.Q., Wang H., Xi B.Y. (2016). Analysis of the role of ultrasonic cleaning in quantitative evaluation of the retention of tree leaves to atmospheric particles: A case study with *Ginkgo biloba*. Sci. Silvae Sin..

[24] Liu J.Q., Cao Z.G., Zou S.Y., Liu H.H., Hai X., Wang S.H., Duan J., Xi B.Y., Yan G.X., Zhang S.W. (2018). An investigation of the leaf retention capacity, efficiency and mechanism for atmospheric particulate matter of five greening tree species in Beijing, China. Sci. Total Environ..

[25] Xu H.M., Cao J.J., Chow J.C., Huang R.J., Shen Z.X., Chen L.W.A., Ho K.F., Watson J.G. (2016). Inter-annual variability of wintertime PM_2.5_ chemical composition in X’’an, China: Evidences of changing source emissions. Sci. Total Environ..

[26] Tao J., Zhang L.M., Engling G., Zhang R.J., Yang Y.H., Cao J.J., Zhu C.S., Wang Q.Y., Luo L. (2013). Chemical composition of PM_2.5_ in an urban environment in Chengdu, China: Importance of springtime dust storms and biomass burning. Atmos. Res..

[27] Galon-Negru G.A., Olariu R.I., Arsene C. (2019). Size-resolved measurements of PM_2.5_ water-soluble elements in Iasi, north-eastern Romania: Seasonality, source apportionment and potential implications for human health. Sci. Total Environ..

[28] Jacobson M.C., Hansson H.C., Noone K.J., Charlson R.J. (2000). Organic atmospheric aerosols: Review and state of the science. Rev. Geophys..

[29] Grosjean D. (1975). Solvent extraction and organic carbon determination in atmospheric particulate matter: The organic extraction organic carbon analyze (OE-OCA) technique. Anal. Chem..

[30] Zhang F. (2013). Studies on the Existing Shrubs of the Road in Changchun and the Dust Eetention Capacity of the Three Shrubs. Master Dissertation.

[31] Chen L.X., Liu C.M., Zou R., Yang M., Zhang Z.Q. (2016). Experimental examination of effectiveness of vegetation as bio-filter of particulate matter in the urban environment. Environ. Pollut..

[32] Xu Y.S., Xu W., Mo L., Heal M.R., Xu X.W., Yu X.X. (2018). Quantifying particulate matter accumulated on leaves by 17 species of urban trees in Beijing, China. Environ. Sci. Pollut. R..

[33] Čeburnis D., Steinnes E. (2000). Conifer needles as biomonitors of atmospheric heavy metal deposition: Comparison with mosses and precipitation, role of the canopy. Atmos. Environ..

[34] Nieminen T.M., Derome J., Helmisaari H.S. (1999). Interactions between precipitation and Scots pine canopies along heavy-metal pollution gradient. Environ. Pollut..

[35] Hong X.L., Yang X.Y., Yang M.Y., Zhong Y.X., Li C., Zhang T., Liu Y.J. (2015). A method of quantifying the retention of PM_2.5_ and other atmospheric particulates by plant leaves. J. Beijing For. Uni..

[36] Przybysz A., Sæbø A., Hanslin H.M., Gawroński S.W. (2014). Accumulation of particulate matter and trace elements on vegetation as affected by pollution level, rainfall and the passage of time. Sci. Total Environ..

[37] Popek R., Gawrońska H., Wrochna M., Gawroński S.W., Sæbø A. (2012). Particulate matter on foliage of 13 woody species: Deposition on surfaces and phytostabilisation in waxes–a 3-year study. Int. J. Phytoremediat..

[38] Song Y.S., Maher B.A., Li F., Wang X.K., Sun X., Zhang H.X. (2015). Particulate matter deposited on leaf of five evergreen species in Beijing, China: Source identification and size distribution. Atmos. Environ..

[39] Wang H.X., Shi H., Li Y.Y., Zhang J. (2013). Seasonal variations in leaf capturing of particulate matter, surface wettability and micromorphology in urban tree species. Front. Env. Sci. Eng..

[40] Wang H.X., Shi H., Li Y.Y., Wang Y.H. (2014). The effects of leaf roughness, surface free energy and work adhesion on leaf water drop adhesion. PLoS ONE.

[41] Zuo N., Wang H.X., Yang Z., Zhong M.T., Shi H., Wang Y.H. (2017). The properties of *Pinus bungeana* captured particulates from atmosphere with different diameters at the breast height. Chin. J. Ecol..

[42] Chen F., Zhou Z.X., Wang P.C., Li H.F., Zhong Y.F. (2006). Green space vegetation quantity in workshop area of Wuhan Iron and Steel Company. Chin. J. Appl. Ecol..

[43] Chen Y., Sui H.D., Feng Z.K., Sun Y.Q. (2009). Comparative analysis on the precisions between two methods for tree height measurement. For. Inventory Plan..

[44] Knoll D., Schreiber L. (1998). Influence of epiphytic micro-organisms on leaf wettability: Wetting of the upper leaf surface of *Juglans regia* and of model surfaces in relation to colonization by micro-organisms. New Phytol..

[45] Gindl M., Sinn G., Gindl W., Reiterer A., Tschegg S. (2001). A comparison of different methods to calculate the surface free energy of wood using contact angle measurements. Colloids Surf. A.

[46] Young T. (1805). An essay on the cohesion of fluids. Philos. Trans. R. Soc. Lond..

[47] Fowkes F.M. (1962). Determination of interfacial tensions, contact angles, and dispersion forces in surfaces by assuming additivity of intermolecular interactions in surfaces. J. Phys. Chem..

[48] Owens D.K., Wendt R.C. (1969). Estimation of the surface free energy of polymers. J. Appl. Polym. Sci..

[49] Fernández V., Sancho-Knapik D., Guzmán P., Peguero-Pina J., Gil L., Karabourniotis G., Khayet M., Fasseas C., Heredia-Guerrero J.A., Heredia A. (2014). Wettability, polarity and water absorption of *Quercus ilex* leaves: Effect of leaf side and age. Plant Physiol..

